# Mechanisms, Mediators, and Pharmacological Approaches Targeting Brain Cholesterol Transport in Alzheimer’s Disease

**DOI:** 10.2174/0113816128411158250909151734

**Published:** 2025-10-01

**Authors:** Martina Ugolotti, Bianca Papotti, Alessandro Trentini, Gianmarco Mola, Carlo Cervellati, Maria Pia Adorni, Francesca Zimetti

**Affiliations:** 1 Department of Food and Drug, University of Parma, 43124, Parma, Italy;; 2 Department of Environmental and Prevention Sciences, University of Ferrara, 44121, Ferrara, Italy;; 3 Department of Translational Medicine and for Romagna, University of Ferrara, 44121, Ferrara, Italy;; 4 Department of Medicine and Surgery, University of Parma, 43125, Parma, Italy

**Keywords:** Alzheimer's disease, brain cholesterol, HDL-like particles, apolipoprotein E4, cholesterol efflux, PCSK9

## Abstract

Cholesterol transport within the brain represents a highly regulated process essential for maintaining neuronal function and central nervous system (CNS) homeostasis. Unlike peripheral tissues, the brain relies on *in situ* cholesterol synthesis, primarily by astrocytes and other glial cells, which supply neurons *via * high-density lipoprotein (HDL)-like particles, identified in the human cerebrospinal fluid (CSF). The major component of HDL-like lipoproteins is the apolipoprotein E (ApoE), whose E4 isoform represents the strongest genetic risk factor for late-onset Alzheimer’s disease (AD). Growing evidence suggests that impaired cholesterol transport contributes to the pathogenesis of various neurodegenerative disorders, particularly AD, a major public health concern due to increasing prevalence and the lack of effective treatments. Indeed, the unconvincing outcomes of the amyloid-targeting monoclonal antibodies underscore the urgency of identifying alternative therapeutic strategies. This review provides a comprehensive analysis of cholesterol transport mechanisms within the brain and their dysregulation in AD by examining the astrocyte-to-neuron cholesterol supply pathways, including endogenous biosynthesis, cholesterol efflux from astrocytes, neuronal uptake, and intracellular processing. Key molecular players involved in each step are discussed, focusing on their roles in AD pathophysiology and potential as therapeutic targets. Furthermore, the review critically evaluates recent preclinical studies exploring pharmacological interventions able to modulate cerebral cholesterol homeostasis. These emerging approaches offer promising alternatives to amyloid-based treatments and may open new perspectives for preventing or mitigating neurodegeneration in AD. By providing an integrated overview of cholesterol transport in the brain, this review highlights novel directions for research and drug development targeting CNS cholesterol metabolism.

## 1 INTRODUCTION

Cholesterol transport in the brain is a peculiar aspect of the cerebral cholesterol metabolism. It represents a complex process that requires the correct interplay of several mediators and is tightly regulated to ensure proper brain functions. Indeed, it is responsible for the correct neuronal supply of cholesterol, derived from astrocytes and other glial cells, and carried by HDL-like particles [1]. As occurs for plasma HDL, brain lipoproteins are assembled through the activity of membrane cholesterol transporters, undergo remodelling mediated by specific enzymes and transport proteins, and finally deliver cholesterol to neurons by a receptor-mediated internalization process [2]. In this respect, an increasing body of evidence has underlined how an alteration of this process is related to different neurodegenerative conditions [3], especially Alzheimer’s disease (AD) [4, 5]. Thus, the identification of specific defects in the different steps of the brain lipoprotein-mediated cholesterol transport can be instrumental in unravelling potential innovative pharmacological targets for novel therapies against AD. In this regard, despite that the clinical relevance of the pharmacological approaches targeting brain cholesterol transport has not been fully proven yet, the few available clinical data seem to be promising. AD pathology is currently a public health issue due to an increasing number of people affected by the disease, a lack of effective treatments in the market, and the unconvincing results of amyloid-β-targeting monoclonal antibodies [6, 7]. Based on these observations, several lines of research are now investigating the individual factors involved in cerebral cholesterol homeostasis as potential novel drug targets to counteract or slow down neurodegeneration [8]. Among these factors, apolipoprotein E (ApoE), which plays an essential role in cholesterol transport in the central nervous system (CNS), is also one of the major genetic risk factors for late-onset AD (LOAD) occurrence, supporting the relationship between an impaired brain lipid metabolism and this pathology [9]. This review aims to provide a detailed overview of the brain cholesterol transport process and its alteration in AD by dissecting out the main steps leading to the supply of astrocyte-derived cholesterol to neurons, namely the endogenous cholesterol biosynthesis, cholesterol efflux from astrocytes, and neuronal cholesterol internalization and processing. The activity of the main mediators of the entire process is discussed, as well as their potential role as pharmacological targets in AD. In addition, the pre-clinical and clinical results of the main pharmacological approaches targeting brain cholesterol transport are listed and critically analyzed. To this end, a literature search was conducted using the PubMed database, prioritizing papers published between 2015 and 2025. The following search strategy was utilized: (Alzheimer’s disease) AND (astrocytes cholesterol biosynthesis) OR (astrocytes cholesterol efflux) OR (CSF HDL) OR (neuronal cholesterol uptake) OR (neuronal cholesterol processing). The literature search was repeated by adding the words “mechanisms” and “pharmacological strategy”. The following studies were considered: (i) *In vitro* studies on both primary and continuous cell lines; (ii) *In vivo* studies on WT or disease-related animal models; (iii) *Ex vivo* studies on biological samples from animal models or humans; and (iv) Clinical studies reporting on molecular observations and pharmacological interventions. Among these, we focused particularly on those employing strong experimental designs and appropriate models to ensure relevance and reliability. Discrepancies or inconsistent findings across studies, if present, have been reported and discussed to enhance the transparency and rigor of the review.

## 2 BRAIN CHOLESTEROL TRANSPORT

Given the essential role of cholesterol in the CNS for correct brain development and functionality, it is fundamental to firmly maintain its cerebral homeostasis, regulating its synthesis, distribution, and excretion [1], as elucidated in Fig. (**1**). Each of these processes seems to be defective in AD, as highlighted in Table **1** and described in the following sections.

### 2.1 Endogenous Cholesterol Biosynthesis

Physiologically, the brain meets its cholesterol need by *de novo* biosynthesis in the CNS, since peripheral cholesterol itself is not able to cross an intact blood-brain barrier (BBB). At the cellular level, the endogenous cholesterol biosynthesis in adult brain mainly involves glial cells, such as oligodendrocytes, microglial cells, and, above all, astrocytes, rather than neurons. In fact, neurons significantly take part in cholesterol biosynthesis during the embryonic and post-natal period, when there is an extensive myelination process; thereafter, adult neurons’ capacity to synthesize cholesterol is significantly reduced and they preferentially rely on astrocytic cholesterol production, except for some brain areas, such as the cortex and the hippocampus, where adult neurons retain some capacity for cholesterol synthesis, also in adulthood [10-12]. Cholesterol synthesis occurs in peroxisomes and endoplasmic reticulum within the cell, and the pathway begins with the condensation of two molecules of acetyl coenzyme A (acetyl-CoA) into hydroxymethylglutaril coenzyme A (HMG-CoA) by the activity of the enzyme HMG-CoA synthase. The rate-limiting step of the process is the conversion of HMG-CoA to mevalonate, a reaction catalysed by HMG-CoA reductase (HMGCR), the well-known pharmacological target of statins [13]. Mevalonate then undergoes a series of reactions to be converted into sterol intermediates (3-isoprenyl pyrophosphate, farnesyl pyrophosphate, squalene) up to the synthesis of lanosterol, where the downstream steps occur following two different biochemical routes: the Bloch or the Kandutsch-Russell pathways [14]. The first biosynthetic pathway mostly characterizes astrocytes’ cholesterol production, employing desmosterol as the main cholesterol precursor and 24-dehydrocholesterol reductase (DHCR24) as the key enzyme. On the other hand, the Kandutsch-Russell pathway is prevalent in adult neurons in the view that there is an increase in lathosterol and 7-dehydrocholesterol as cholesterol precursors, as well as a lower DHCR24 and CYP51 activity, suggesting a different enzymatic pool between the two pathways [12, 15]. Although some studies have not highlighted any differences in terms of HMGCR mRNA levels in all brain areas by comparing AD patients and control subjects [16], most of them have demonstrated an involvement of HMGCR in AD pathology. Alterations of this biosynthetic process are indeed associated with neurodegenerative disorders, as described in a study where patients with AD showed a decrease in HMGCR mRNA and an increase in sterol regulatory element-binding protein 2 (SREBP2) expression selectively in neurons, while astrocytes were not affected [17]. As observed by Cao *et al*., HMGCR plays an important role in the maintenance of brain structure. Indeed, the rs3846662 HMGCR polymorphism has been demonstrated to affect the correct entorhinal cortex and hippocampus volume, possibly leading to atrophy; notably, this effect showed a correlation with the progression of the pathology [18]. To further support the involvement of HMGCR in AD pathology, it was also observed that HMGCR G-negative polymorphism rs3846662 exerted a protective effect against the onset of AD, also mitigating the ApoE4-related risk in mild cognitive impairment (MCI) patients [19]. Beyond its primary activity in mediating cholesterol biosynthesis, HMGCR exerts many other biological functions, being involved in oxidative stress, neuroinflammation, microglial proliferation, and amyloid β (Aβ) deposition, typical hallmarks of AD [20]. HMGCR is not the only cholesterol biosynthetic enzyme that exhibits a relationship with neurodegeneration. Indeed, the inhibition of DHCR24, both through an inhibitor compound and with a siRNA-based approach, was associated with cholesterol depletion, resulting in a reduction in plasticity and functionality in neuronal cells, as well as a significant accumulation of Aβ and derangement of cognitive performance in a rat model [21]. In line with these observations, it has been demonstrated that mice treated with a high-fat diet (HFD) showed a downregulation of DHCR24 expression in the hippocampus and brain injury. Consistently, *in vitro* cholesterol overload caused neuronal apoptosis *via* DHCR24 expression downregulation, and this result was reversed by DHCR24 upregulation, corroborating the crucial role of this enzyme in neurodegeneration [22].

### 2.2 Cholesterol Efflux from Astrocytes

Brain cholesterol, after being endogenously synthesized, is then exported from the cell through active pathways involving ATP-binding cassette (ABC) transporters and incorporated into HDL-like particles in the extracellular space [23]. In this context, both the capacity of cells to release cholesterol and the ability of HDL-like particles in the extracellular environment to accept cellular cholesterol are discussed below as two distinct steps of this process.

#### 2.2.1 Cellular Ability to Release Cholesterol

The efflux of newly synthesized cholesterol in the extracellular space mainly occurs through the ABC transporters. Among these, ABCA1 and ABCG1 are the major members of this family in the CNS, but other proteins may be involved, such as ABCA7 and ABCG4 [24]. This family of transporters is under the transcriptional regulation of liver X receptor (LXR); accordingly, agonists of this specific nuclear receptor led to an upregulation of ABC transporters’ expression [25]. These transporters promote the efflux of unesterified cholesterol and phospholipids to glial-derived apolipoproteins by forming HDL-like particles, lipoproteins similar in size and density to plasma HDL [4]. Specifically, ABCA1 is the major contributor to cholesterol efflux into lipid-free or lipid-poor ApoE and ApoA-I, while ABCG1 is responsible for further HDL lipidation. This process is tightly regulated, as it plays a critical role in maintaining cholesterol homeostasis in the brain. Dysregulation of cholesterol transporters and their impaired function have been implicated in the pathophysiology of neurodegenerative disorders, particularly AD [26, 27]. In individuals with the ApoE4 variant, a well-established genetic risk factor for AD, ABCA1 is trapped in lysosomes, reducing cholesterol efflux and leading to cholesterol accumulation and cellular senescence [28]. This observation goes along with another evidence demonstrating a poor ABCA1 membrane recycling as well as its low ability to properly lipidate ApoE in primary mouse astrocytes with a targeted replacement of ApoE with human ApoE4 (ApoE4-TR mice) [29]. In addition, ABCA1 further impacts Aβ metabolism by suppressing Aβ precursor protein (APP) proteolysis and promoting Aβ clearance, while ABCG1 reduces Aβ production [30, 31]. In this regard, an association has been found between a rare loss-of-function mutation in ABCA1 and the risk of early-onset AD; this could be related to a reduced lipidation of ApoE-containing lipoproteins as well as an increase in Aβ deposition, as the lack of ABCA1 affects APP activity and Aβ clearance [32-35]. On the other hand, a detrimental effect of Aβ aggregates on ABCA1 function has been reported: without affecting ABCA1 expression, Aβ inhibits its functionality by obstructing the transporter itself and thereby reducing cell cholesterol efflux [15, 36]. Another cholesterol transporter relevant to AD is ABCA7, mainly expressed in microglia and oligodendrocytes, and it has been shown to play a role in the modulation of phagocytosis of Aβ aggregates, thus contributing to their degradation and preventing their deposition [37]. Furthermore, single-nucleotide polymorphisms (SNPs) in the ABCA7 gene have been linked to an increased AD risk, since they have been found in up to 7% of AD patients [38]. ABCG4, beyond mediating desmosterol and cholesterol efflux, is also responsible for Aβ efflux, regulating its clearance from the brain. In addition, both ABCG4 and ABCG1 were found to be able to inhibit γ-secretase activity in an AD cell model, lowering Aβ levels [39].

#### 2.2.2 HDL-like Particles' Ability to Accept Cellular Cholesterol

After cholesterol has been released, it is incorporated into lipoproteins similar in size and composition to plasma HDLs, called, for this reason, “HDL-like particles”. These particles are assembled *in situ* and enriched with apolipoproteins and accessory proteins that can modulate their function. The most important function of brain HDL is to carry cholesterol through cerebrospinal fluid (CSF), therefore guaranteeing neuronal cholesterol supply and neuronal function [2]. This is possible mostly because of the presence of specific apolipoproteins that drive HDL lipidation and specific interactions with neurons. ApoE is the main apolipoprotein secreted from astrocytes by ABCA1 in the form of nascent ApoE-containing HDLs that are assembled with unesterified cholesterol and subsequently lipidated. However, there are also other apolipoproteins assembled in HDL-like particles, such as ApoA-I and, to a lesser extent, ApoJ, ApoC1, ApoH, and ApoM [40, 41]. The ability of HDL to correctly carry and exchange lipids in the CNS is considered fundamental for brain functionality; defects in HDL function are indeed strongly associated with the development of neurodegenerative disorders, such as AD. Specifically, a dysregulation of HDL-like particles functionality in accepting cellular cholesterol, measured as cholesterol efflux capacity (CEC), can lead to neurodegeneration, as extensively described by a wide range of studies [42]. In this regard, we previously found reduced CSF CEC from astrocytes in AD patients compared to cognitive normal subjects [15, 43]. In agreement with this evidence, in another study, we observed altered CSF CEC in AD patients, specifically evaluating the ABCA1- and ABCG1-mediated contribution (-73% and -33%, respectively) [44]. These observations have been found to be in line with other studies that have shown an impairment of CSF ABCA1-mediated cholesterol efflux since the early stages of the disease [45]. Brain HDLs, after the promotion of cholesterol efflux, may undergo a process of maturation in the extracellular space, leading to the shift from discoidal to spherical HDL, attributed to the activity of remodelling enzymes, manly lecithin cholesterol acyltransferase (LCAT), but also cholesteryl ester transfer protein (CETP) and phospholipid transfer protein (PLTP), which are responsible for changes in HDL lipid composition and size [46]. Indeed, another feature of HDLs that seems to influence their functionality and is associated with AD pathology is their size. Also, the presence of different apolipoproteins on the HDL surface is fundamental for efficient cholesterol transport. In this regard, a growing body of evidence has highlighted a different lipidation state of ApoE according to its isoforms, showing a reduced lipidation rate with ApoE4 compared to ApoE2 and ApoE3, as a result of the lower ability to promote cholesterol efflux [47-49]. This detrimental effect could be mechanistically explained by a dysfunction in ApoE4 aminoacidic structure, leading to an interaction between the amino- and the carboxy-terminal domains of the protein that reduces the ability of this isoform to correctly mediate cholesterol efflux from cells [50]. In addition, a correlation between ApoA-I and AD has been observed. It was found that APP/PS1 mice with a complete ApoA-I deficiency showed increased levels of Aβ deposition, particularly in the cortex, and an enhanced inflammation compared to mice hemizygous for ApoA-I [51]. Finally, ApoJ, also known as clusterin, has been demonstrated to correlate with neurodegeneration. Indeed, CSF ApoJ levels were significantly lower in patients with MCI compared to controls and were associated with a reduced CSF cholesterol efflux capacity [52].

#### 2.2.3 HDL-like Accessory Proteins

The protein composition of plasma HDL is highly diverse, extending well beyond the commonly dominant proteins ApoA-I and ApoA-2 [53, 54]. A recent review analyzed proteomics studies from various laboratories using different isolation methods and identified 251 consensus HDL-associated proteins [55]. Some of these proteins are known to play significant roles in inflammation, redox mechanisms, coagulation, immunity, and lipid transport and metabolism. Together with ApoA-I, ApoA-2, and phospholipids, they modulate the pleiotropic activities of the lipoprotein, primarily aimed at protecting against atherogenesis [55, 56]. However, it is well-documented that adverse conditions, such as excessive inflammation, can disrupt HDL protein composition. This disruption can ultimately reverse HDL's function from being athero-protective to pro-atherogenic [57]. Changes in the level of some HDL accessory proteins, a group of proteins associated with the lipoprotein in addition to the constitutive apolipoproteins, significantly contribute to this functional change. Notable among them are paraoxonase-1 (PON1) and PON3 [58, 59], lipoprotein phospholipase A2 (Lp-PLA2) [60], myeloperoxidase (MPO) [61, 62], glutathione peroxidase 3 (GPx-3) [56], serum amyloid A protein (SAA) [56, 63], LCAT [64], and CETP [65]. These proteins display various functions in biology and pathology and are distributed differently across the complex HDL family, as described in various reviews [53, 55, 56]. Currently, there is no clear and direct evidence showing that any of these proteins is present in CSF, associated with HDL-like particles circulating in the brain vessels, while CSF GPx3, MPO, LCAT (most likely secreted by astrocytes) [46, 66], and PON1 have been widely documented in the human or animal brain [67-71]. Although some studies have reported detectable amounts of these proteins in CSF, the findings are sparse. For instance, Fernández-Espejo recently found similar levels of the pro-oxidant MPO concentrations and activity in the CSF of healthy controls (total sample, n=64) and patients with Parkinson’s disease [72]. Conversely, our recent analysis did not detect measurable levels of this pro-oxidant protein in the CSF of cognitively healthy individuals and AD patients (n=70). Similarly, GPx3 was not found in human samples, although it was detected in rats in one study [73]. It cannot be ruled out that using more sensitive analytical approaches, such as HPLC or mass spectrometry, could be more successful in detecting these proteins in this biological fluid. On the contrary, PON1 activity has been detected in human CSF in three studies using the same spectrophotometric method employed for plasma detection [74, 75]. PON1 is a member of the paraoxonase family, which also includes PON2 and PON3. These calcium-dependent enzymes exhibit various properties, such as antioxidant, anti-inflammatory, anti-microbial, and xenobiotic detoxifying (specific to PON1) [59]. While PON2 is found intracellularly (and is present in relatively high amounts in brain tissue), PON1 and PON3 are associated with the surface of circulating HDL [56, 59]. The most relevant role of PON1 is the antioxidant defence of LDL and macrophages. These cells possess natural binding sites for PON1-HDL through which the hydrolase contributes to counteracting intracellular oxidative stress and promoting cholesterol efflux to HDL [76, 77]. *In vivo* studies involving an AD mouse model have clearly shown PON1 to be present in the brain [78, 79], with lower concentration/activity observed in transgenic mice compared to wild-type mice. Interestingly, no PON1 mRNA was detected in this tissue, suggesting that the protein may originate from peripheral sources. Conversely, minimal traces of PON1 mRNA have been detected in the human brain [80]. Although substantial evidence links low plasma/serum PON1 levels to AD, only one of our studies has investigated the enzyme's activity in the CSF of patients with this form of dementia [81]. We observed a trend, nearing statistical significance, indicating lower PON1 levels in AD patients compared to cognitively healthy controls. Notably, CSF PON1 showed a strong correlation with ApoA-I in the same biological fluids (r = 0.685, *p* < 0.001), suggesting PON1 in CSF to also bound to ApoA-I, and thus to HDL-like particles. Another evidence of PON1-HDL-like particles was the observation that PON1 lost its activity and structural integrity when it did not bind to ApoA-I and HDL phospholipids [81]. The unresolved questions primarily focus on PON1's role and influence in the brain. As our earlier data indicate, this protein may maintain a close physical association with ApoA-I within HDL-like particles, similar to its interaction with peripheral HDL. Since multiple studies have supported the idea that ApoA-I and PON1 work together to enhance HDL's antioxidant properties, it can be hypothesized that this functional cooperation may extend to the brain as well. Supporting this, we observed an inverse correlation between PON1 activity and levels of 4-hydroxy-nonenal (4-HNE), a reactive aldehyde generated by lipid peroxidation, in the hippocampal region of AD transgenic mice [79]. Based on these findings, it is plausible to hypothesize that the interaction between PON1 and ApoA-I may influence cholesterol trafficking in the brain, as seemingly occurs in the periphery. Indeed, our work, alongside studies by others, suggests that these proteins may cooperatively regulate cholesterol efflux from macrophages. More specifically, we found PON1 activity to be positively associated with both aqueous and scavenger receptor class B type 1 (SR-BI)-mediated cholesterol efflux pathways, with this association strongly influenced by ApoA-I levels. These findings were consistent with an *in vitro* study showing PON1 to interact with plasma membrane lipid rafts of macrophage membranes, thereby mediating cholesterol efflux [82]. Moreover, overexpression of human PON1 in mice was shown to improve the capacity of HDL to mediate cholesterol efflux [76].

### 2.3 Neuronal Cholesterol Internalization and Cholesterol Processing

#### 2.3.1 Cholesterol Internalization into Neurons

Astrocyte-derived cholesterol is delivered to neurons in the last step of the brain cholesterol transport. Notably, apolipoproteins present on HDL-like particles can interact with specific membrane receptors, allowing neuronal cholesterol internalization, essential to provide the neurons with a cholesterol supply to guarantee correct brain viability and functionality [4]. For this reason, the presence of specific neuronal receptors is finely regulated both at the transcriptional and post-transcriptional levels. A growing body of evidence points to how an impairment of cholesterol delivery to neurons is associated with neurodegeneration. In this regard, a study measuring CSF HDL-like particle-mediated neuronal cholesterol uptake showed this process to be significantly reduced in AD patients compared to the control group [83]. Notably, neuronal cholesterol internalization is mainly mediated by a wide family of receptors, of which low-density lipoprotein receptor (LDLR), apolipoprotein E receptor 2 (ApoER2), and low-density lipoprotein receptor-related protein 1 (LRP1) are the most relevant. An alteration of cholesterol delivery to neurons is very likely due to defects in the receptors responsible for this process. In line with this concept, mice lacking LDLR exhibited significant memory impairment [84-86], which was more pronounced in middle-aged mice, likely due to an exacerbation of neuronal apoptosis and possibly a reduction in neuronal cholesterol supply [87, 88]. In agreement with this, Perkovic and colleagues reported a heterozygous mutation of LDLR variant rs140241383 associated with AD in a 44-year-old man [89, 90]. Beyond receptor expression, another relevant aspect of neuronal cholesterol internalization is the proper interaction between apolipoproteins, mainly ApoE, and neuronal receptors, such as LDLR. After this interaction, an ApoE-LDLR complex is formed and subsequently internalized, leading LDLR to an intracellular recycling pathway that regulates its turnover. Interestingly, an *in silico* study suggested that due to the difference in the pH dependence of the LDLR-ApoE association, a different response might be predicted, with ApoE3-associated LDLR being recycled back to the plasma membrane, while ApoE4-LDLR remaining trapped in the late endosome for degradation [91]. In this context, the ApoER2 pathway seems to be strongly linked to the risk of AD. In particular, the disruption of the ApoER2-disabled-1 (ApoER2-Dab1) pathway, which is implicated in memory regulation, was observed to be associated with an increase in phospho-tau (p-tau) formation, thereby worsening neurodegeneration [92]. Additionally, alternative splicing combinations across the ApoER2 gene were identified as differentially related to the risk of the disease. Indeed, upon analysing the transcriptome of parietal cortex and hippocampus of AD patients, several ApoER2 isoforms were found to be present and thus associated with synaptic dysfunction and neurodegeneration [93]. In this regard, a study demonstrated the levels of ApoER2 to be lower in AD patients compared to control subjects, especially in the ε4 carriers, corroborating the implication of this pathway in the pathology. Moreover, in the same study, it was observed that in CSF of AD patients, there was an alteration of reelin fragments, probably due to a different cleavage both at the N- and C-terminal site [94]. Reelin is a glycoprotein present in the CNS that, beyond regulating the early brain development and synaptogenesis, binds to the LDLR family, especially ApoER2. For this reason, it has been associated with the modulation of brain cholesterol metabolism and brain disorders, including Alzheimer’s disease [95]. Indeed, it has been reported that a patient carrying a gain-of-function mutation of *RELN*, the gene encoding reelin, developed resilience against presenilin-linked AD. These results were confirmed by a study conducted on a mouse model, demonstrating also the activation of reelin pathway to inhibit tau phosphorylation, amyloidogenic APP processing, and Aβ generation [96]. LRP1 is another essential player in the regulation of cerebral cholesterol homeostasis, and it exerts many other functions in the brain that are strongly associated with AD pathology. Besides acting as a receptor for ApoE, LRP1 is indeed present in the pericytes of the BBB, where it is responsible for the interchange of soluble Aβ aggregates between the brain and the periphery, contributing to Aβ clearance [97]. Moreover, as demonstrated by an *in vivo* study on APP/PS1 transgenic mice, brain LRP1 silencing leads to an exacerbation of AD pathology in terms of Aβ deposition, neuroinflammation, synaptic and neuronal loss, as well as spatial cognitive performance. In addition, LRP1 silencing enhances pro-inflammatory cytokine production *via* toll-like receptor 4 (TLR-4)/nuclear factor-kappa B (NF-kB)/mitogen-activated protein kinase (MAPK) signalling pathway, suggesting an involvement in the neuroinflammatory process [98]. The expression of membrane receptors that mediate neuronal cholesterol internalization might undergo modulation by specific factors, among which proprotein convertase subtilisin/kexin type 9 (PCSK9) and inducible degrader of LDLR (IDOL) are the most important, with partially redundant mechanisms of action. PCSK9, beyond its well-established role in the handling of plasma cholesterol levels [99], plays several roles in the CNS, being involved in neurogenesis, neuronal differentiation, neuroinflammation, as well as in the modulation of cerebral cholesterol metabolism, thus suggesting an involvement in the pathogenesis of AD [100, 101]. Supporting this hypothesis, we previously reported higher CSF PCSK9 levels in AD than in non-AD subjects, and in AD patients, the highest levels were retrieved among the ApoE4 isoform carriers [102, 103]. Consistently, Picard *et al*. reported significantly elevated PCSK9 mRNA and protein levels in the frontal cortices of AD patients compared to control subjects [104]. Mechanistically, we previously demonstrated PCSK9 to mediate the degradation of neuronal LDLR and ApoER2, thereby resulting in decreased ApoE-mediated cholesterol uptake, eventually leading to a reduction in intracellular cholesterol, a potentially detrimental effect. Furthermore, PCSK9 enhanced Aβ-fibrils-induced neurotoxicity [105] and astrocyte-derived secretion of pro-inflammatory cytokines [106]. In line with this evidence, we recently demonstrated that the genic ablation of PCSK9 in an *in vivo* mouse model of severe AD (5XFAD mice) resulted in a significant improvement in cognitive performance, together with a reduction in cortico-hippocampal Aβ plaque burden and microglial and astrocyte reactivity [106]. Altogether, this evidence suggests that PCSK9 may influence several processes traditionally associated with AD pathogenesis. However, a recent study conducted on WT mice with a neuron-specific PCSK9 deletion showed impaired cognitive function and altered hippocampal synapse morphology as a consequence of the lack of post-translational regulation of ApoER2 exerted by PCSK9 [107]. This data, although apparently conflicting, might suggest that PCSK9's detrimental effects may be specifically associated with a pathological state (*i.e*., in experimental neurodegeneration models), while playing a non-negative role in physiological contexts. Nevertheless, this evidence sheds light on the complex PCSK9 biology in the CNS, requiring further investigation. Another post-transcriptional regulator of the ApoE receptor family present also at a central level is IDOL, an E3 ubiquitin ligase, responsible for ubiquitylation and subsequent lysosomal degradation of the LDLR, thereby reducing the internalization of cholesterol [108]. Consistently, IDOL has been demonstrated to be involved in the process of memory formation, suggesting a role in AD [109]. In this regard, Choi and colleagues observed increased brain LDLR and decreased Aβ burden and neuroinflammation in transgenic APP/PS1; IDOL-/- mice. Their findings identified IDOL to be among the most relevant physiological regulators of cerebral LDLR, involved in the modulation of ApoE-containing HDL-like particles clearance and Aβ plaques deposition [110, 111].

#### 2.3.2 Neuronal Cholesterol Processing

Due to the limited exchange between plasma and CNS cholesterol, a proper balance between cholesterol biosynthesis and metabolic clearance appears to be critical; notably, both cholesterol depletion and excess cholesterol accumulation are pathologically related to AD aetiopathology [112]. A significant part of cerebral cholesterol content undergoes extensive metabolization, maintaining a tight balance between its synthesis, cellular internalization, and elimination. For instance, the main cholesterol elimination pathway in neurons involves its hydroxylation by the cytochrome P450 enzymes, mostly CYP27A1, CYP7A1, and CYP46A1, thereby generating 27-hydroxycholesterol and 24S-hydroxycholesterol (24HC), respectively [113]. Interestingly, CYP46A1, highly expressed in the pyramidal neurons of the cortex and hippocampus, in the hippocampal and cerebellar interneurons, and in the nucleus striatum [114], accounts for the production of 98-99% of cerebral 24HC, therefore representing the major physiological route for excess cholesterol elimination, being able to readily diffuse out of cell, cross BBB, and enter the systemic circulation for its final biliary elimination [113, 115]. CYP46A1 enzyme has therefore drawn considerable interest in AD due to its critical role in converting cholesterol to the 24HC neuroactive oxysterol. A huge amount of data has examined the role of 24HC in AD pathogenesis, highlighting its activity as a double-edged sword. Indeed, controversial evidence has been reported, with either significantly lower CYP46A1 cerebral expression and thus 24HC generation observed in AD patients [116-120], or increased 24HC levels correlating with the disease [103, 121-124]. Taken together, this evidence points out the complexity of this regulatory pathway, in which an increase in 24HC levels might be the result of ongoing active neuronal damage, typical of the early AD phase, with the release of total free sterols, while in the more advanced stages of AD, 24HC might decline due to the loss of CYP46A1-expressing neurons in a context of severe atrophy [125], thus further complicating the interpretation of the data as a whole. In neuroblastoma cells, 24HC reduced intraneuronal accumulation of hyperphosphorylated tau protein [126, 127]. Similarly, 24-HC also reduced Aβ production by inhibiting intracellular trafficking of amyloid precursor protein [128, 129].

## 3 PHARMACOLOGICAL MODULATION OF BRAIN CHOLESTEROL TRANSPORT

**Fig. (2):**
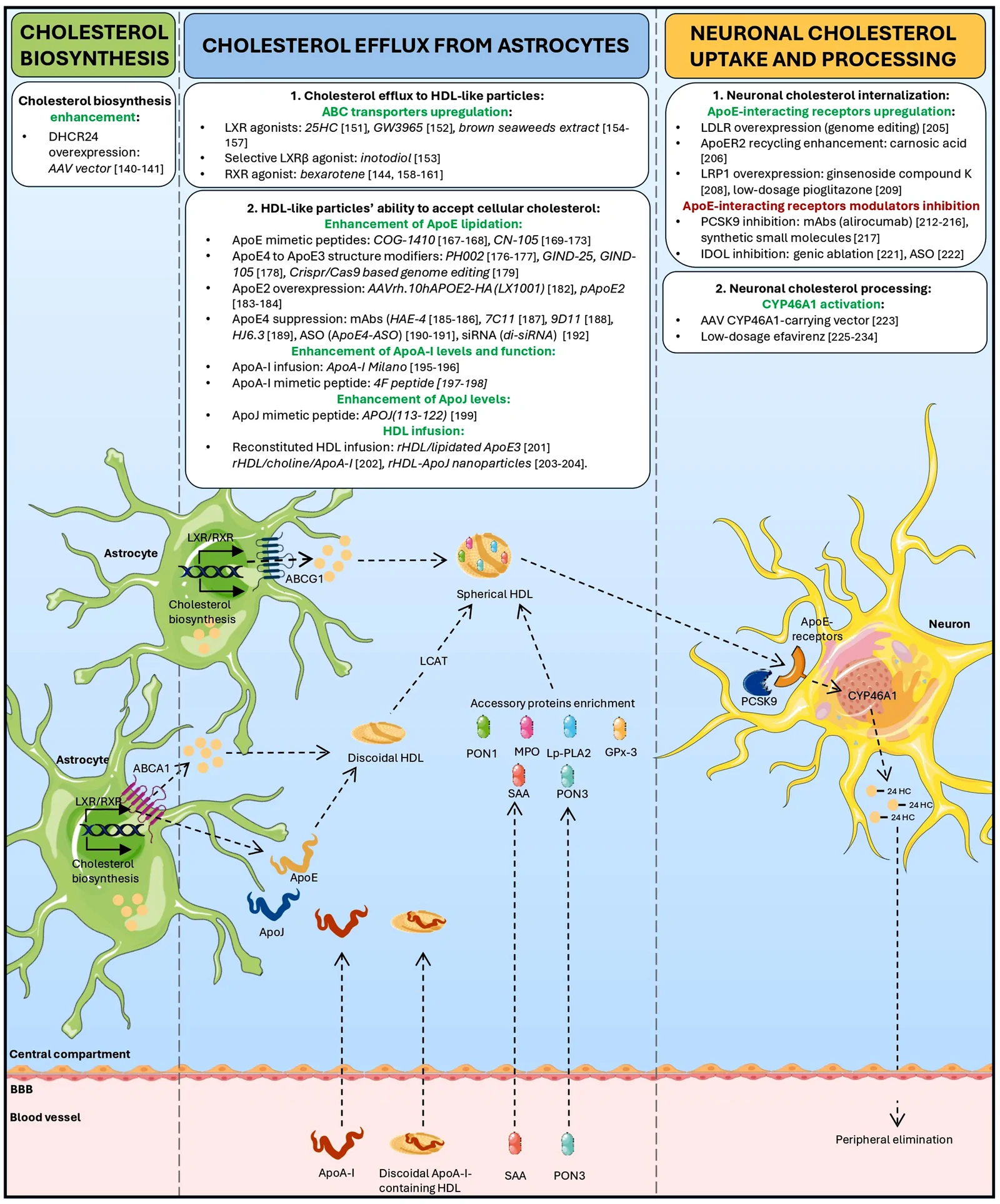
Pharmacological approaches modulating brain cholesterol transport. Several experimental approaches have been proposed to target the specific cholesterol homeostasis alterations reported in AD. These strategies primarily aim to (I) enhance endogenous cholesterol biosynthesis and (II) promote cholesterol efflux from astrocytes by stimulating the cellular ability to efflux cholesterol through ABC transporters, as well as enhancing HDLs’ ability to accept cellular cholesterol. Finally, several pharmacological approaches have explored the possibility of modulating neuronal cholesterol uptake and processing by (III) enhancing ApoE-interacting receptor-mediated neuronal cholesterol internalization, as well as (IV) intraneuronal CYP46A1-mediated cholesterol metabolization. The picture has been created by combining images from Smart Sevier Medical Art (https://smart.servier.com) accessed on 10.04.25. Sevier Medical Art by Sevier is licensed under a Creative Commons Attribution 3.0 Unported License (https://creativecommons.org/licenses/by/3.0/). **Abbreviations**: AAV, Adeno-associated virus; ABCA1, ATP-binding cassette transporter A1; ABCG1, ATP-binding cassette transporter G1; ApoA-I, Apolipoprotein A-I; ApoE, Apolipoprotein E; ApoJ, Apolipoprotein J; ApoER2, Apolipoprotein E receptor 2; ASO, Anti-sense oligonucleotide; BBB, Blood-brain barrier; Crispr/Cas9, Clustered regularly interspaced short palindromic repeats-associated protein 9; CYP46A1, Cytochrome P450 46A1; DHCR24, 24-dehydrocholesterol reductase; GPx-3, Glutathione peroxidase 3; HDL, High-density lipoprotein; 24OH, 24-hydroxycholesterol; 25HC, 25-hydroxycholesterol; IDOL, Inducible degrader of LDL receptor; LDL, Low-density lipoprotein; LDLR, Low-density lipoprotein receptor; Lp-PLA2, Lipoprotein-associated phospholipase A2; LRP1, Low-density lipoprotein receptor-related protein 1; LXR, Liver X receptor; MPO, Myeloperoxidase; PCSK9, Proprotein convertase subtilisin/kexin type 9; PON1, Paraoxonase 1; PON3, Paraoxonase 3; RXR, Retinoid X receptor; siRNA, Small interfering RNA; SAA, Serum amyloid A.

As described in the previous sections and Table **1**, a growing body of evidence points to detrimental alterations in cholesterol homeostasis in the AD brain. Therefore, pharmacological modulation of cerebral cholesterol homeostasis has gained increasing interest as a potential therapeutic strategy in AD-related neurodegeneration, with the aim of restoring or potentiating specific processes related to the cerebral cholesterol machinery that appears to be defective in AD. In the following paragraphs, we critically discuss the challenges and opportunities in the development of cerebral cholesterol modulating therapy, highlighting the potential contribution of the most relevant experimental approaches (Fig. **2**).

### 3.1 Modulation of Cholesterol Biosynthesis

As already described, cholesterol biosynthesis is tightly involved in neurodegeneration processes, particularly AD, and for this reason, biosynthetic enzymes responsible for its production have been examined for their role as potential pharmacological targets to treat this pathology [17]. In this regard, statins, well-known drugs targeting HMGCR and widely used to treat hypercholesterolemia, have been extensively studied for their potential effects in the CNS, in light of their lipophilicity allowing BBB crossing. However, the impact of statins on cerebral function is still under investigation. On one hand, it was reported that HMGCR inhibition can have detrimental effects, as the treatment with lovastatin caused an impairment in the neuritic network in rat neurons, possibly linked to an alteration of the microfilament and microtubule [131]. This result was confirmed by other studies showing the activation of neuronal cell death pathways after the inhibition of HMGCR and the cholesterol biosynthetic process [132]. On the other hand, it was demonstrated that statins can also exhibit interesting protective effects on the brain, and this may be likely due to their several pleiotropic effects [133]. Remarkably, simvastatin has been demonstrated to exert beneficial effects in reducing the risk of developing neurodegenerative disorders, although the underlying mechanisms are still unclear [134]. In general, the inhibition of HMGCR seems to restore mitochondrial functionality acting through nuclear factor erythroid 2-related factor 2 (Nrf2)-dependent pathway, as observed in a rodent model of AD treated with atorvastatin. Indeed, rats that received the statin showed an enhancement in spatial and long-term memory, together with an amelioration of the Aβ_1-42_-induced cognitive decline [13]. In agreement with this observation, an *in vitro* study conducted on neuroblastoma cells highlighted a protective role of atorvastatin against Aβ_1-42_-induced neurotoxicity through the enhancement of expressions of both sestrins and sirtuins, involved in the regulation of apoptosis and antioxidant activity [135]. Several meta-analyses and clinical studies have investigated the possible correlation between the use of statins and the incidence of Alzheimer’s disease; however, the results have not been able to clarify this issue [136-139]. The potential beneficial effects of statins seem to be likely counterbalanced by the drawbacks of HMGCR inhibition in the brain, resulting in a net negligible effect. Another crucial mediator of cerebral cholesterol homeostasis that has been associated with neurodegeneration and AD is DHCR24. DHCR24 has been demonstrated to exhibit a beneficial effect on the microglial inflammatory response by reducing microglial polarization. Indeed, Zu *et al*. observed that mouse microglial cells BV-2 treated with the peptide Aβ_25-35_ underwent a transition of microglial phenotype from M2 to M1, activating the inflammatory response. However, they could observe that when DHCR24 was overexpressed following lentiviral transfection, the inflammatory activation was reversed due to the activation of protein kinase B/glycogen synthase kinase-3β (Akt/GSK3β) signalling pathway [140]. Other *in vivo* experiments have supported the role of DHCR24 as a potential pharmacological target in AD, highlighting the positive effect of DHCR24 on cognitive performance, apparently linked to the modulation of cerebral cholesterol levels. For instance, 5XFAD mice showed reduced cholesterol levels as compared to control mice, but after treatment with adeno-associated virus carrying the DHCR24 gene into the hippocampus, DHCR24 overexpression alleviated the observed cholesterol loss. This effect was paralleled by an improvement in cognitive performance and a reduction in Aβ plaque deposition, synaptic injuries, autophagy, and apoptosis [141]. This evidence suggests that it is possible to reverse neurodegeneration progression, considering cholesterol biosynthetic enzymes as a potential pharmacological target that could ameliorate different factors related to the pathology.

### 3.2 Modulation of Cholesterol Efflux

#### 3.2.1 Modulation of Cholesterol Efflux to HDL-like Lipoproteins

##### 3.2.1.1 ABC Transporters Targeting Applications

As described above, the first step of brain cholesterol transport consists of cholesterol efflux from astrocytes. Many studies have highlighted the importance of ABC transporters mediating this specific process and pointed to them as potential pharmacological targets. Indeed, there are different therapeutic approaches under development, aiming at upregulating ABC transporters, including small molecules able to trigger their activity, such as sterol derivatives, oligonucleotides [29, 142] and small peptides [143, 144], as well as stabilizers that prolong their presence on cell membranes [145, 146], or molecules that can promote their expression, such as LXR agonists [147, 148]. One of the proposed strategies is to exploit the multitarget binding site of pan-ABC transporter inhibitors to develop a novel ABC activator able to ameliorate cholesterol transport and promote Aβ clearance through BBB, a process mediated also by these transporters [149]. In view of the strong involvement of ABC transporters in the pathogenesis and progression of AD, recent *in silico* studies have tried to pave the way for the development of therapeutics addressing ABC transporters by identifying essential pharmacophore features that may represent a treatment for AD [150]. Another way to enhance the expression of ABC transporters on cell membranes and strengthen their activity in promoting cholesterol efflux is by activating their transcriptional receptor LXR. In this regard, Cashikar and colleagues reported the treatment of cultured astrocytes with 25-hydroxycholesterol (25HC) to be associated with increased ApoE secretion as a result of higher *Abca1* expression following LXR stimulation, thus potentially promoting astrocyte cholesterol efflux; in mouse astrocytes-expressing human ApoE3 or ApoE4, 25HC induced extracellular ApoE3 better than ApoE4 [151], further reinforcing the hypothesis associated with an impaired ApoE4-mediated cholesterol efflux capacity. Moreover, as observed by Litvinchuk *et al*. on a mouse model of tauopathy, the administration of 40 mg/kg of GW3965 with the diet for 6 months led to an increased cholesterol efflux from glial cells, thus reducing tau pathology, neurodegeneration, as well as astrocyte and microglial activation [152]. To avoid hepatic lipogenesis caused by pan-LXR agonists, some selective LXRβ agonists are under investigation as a potential approach for the treatment of AD. For instance, an *in vivo* study reported the beneficial effects of the treatment with inotodiol (CE9A215) on the 3xTg-AD mouse model with respect to AD-related parameters. The treatment induced the expressions of ABCA1, ApoE, and SREBP-1c in mice brains, which resulted in a significant reduction in Aβ deposition, tau phosphorylation, and neuroinflammation in the hippocampus, showing prophylactic and therapeutic effects on AD pathology [153]. Another approach involved synthesizing non-lipogenic ABCA1 inducers, which increased ABCA1 expression and ApoE lipidation and ameliorated AD phenotype in an AD mouse model expressing human ApoE3/ ApoE4 [25]. Moreover, it was also observed in both *in vitro* and *in vivo* studies that the activation of LXR by extracts of brown seaweeds, enhancing ABCG1- and ABCA1-mediated cholesterol efflux, could have a potential application in AD [154-157]. A retinoid X receptor (RXR) agonist, bexarotene, showed beneficial effects on cognitive performance in AD mouse models [144, 158-161]. However, the clinical relevance of this pharmacological approach has not been fully proven yet. In this regard, a case study reported that bexarotene treatment in MCI patients led to an amelioration of memory performance and tau phosphorylation [162], while the treatment of moderate AD patients led to increased amyloid clearance only in ApoE4 non-carriers. Further investigation is necessary to determine whether ABC transporters are a valid target for novel therapies against AD [144, 158-161].

#### 3.2.2 HDL-like Particles' Ability to Accept Cellular Cholesterol

##### 3.2.2.1 ApoE-targeting Experimental Approaches

A growing body of evidence has strongly pointed to an altered ApoE4-related cholesterol metabolism, with lower secretion of ApoE itself and reduced ApoE4 lipidation [148], together with modifications in cholesterol synthesis and efflux and uptake genes, thus potentially negatively affecting astrocyte-to-neuron cholesterol delivery [163]. Therefore, several pharmacological strategies are currently under investigation, aiming to reduce the detrimental effects associated with ApoE4 while preserving and/or enhancing the fundamental role of ApoE in brain cholesterol transport. The enhancement of ApoE lipidation may favorably impact AD pathology, considering its critical role in cerebral lipid transport, which is essential for neuronal sustenance, that has been reported to be altered in AD [105, 164]. In this regard, the manipulation of lipid nanocarriers has indeed emerged as a promising strategy to enhance astrocytes-to-neurons cholesterol delivery. By employing lipid nanodisks containing ApoE-derived peptides, Tanaka and colleagues provided proof of concept about the possibility to amplify the cerebral bioavailability of lipidated ApoE [165]. In addition, the enrichment of the nanocarriers with 1-palmitoyl-2-oleoyl-sn-glycero-3-phosphocholine content, might selectively enhance the BBB permeability, thus improving access to the CNS [166]. Another experimental approach being tested involves the use of ApoE mimetic peptides, characterized by low molecular weight, which enhances their ability to cross the BBB. Among these, the administration of the ApoE mimetic peptide COG-1410 (aminoacidic residues 138-149 with amino-iso-butyric acid substitutions at positions 140 and 145 to increase stability) improved spatial learning and memory, reduced intracerebral Aβ deposition and reactive astrocytes in the dentate gyrus of hippocampus, and caused a significant increase in the brain-derived neurotrophic factor (BDNF) in APP/PS1 tg mice [167]. Similar results were obtained in other AD mouse models, such as the CVND-AD [SwDI-APP/NOS2(-/-)] tg mice [168]. Similarly, the early administration of CN-105, a small ApoE mimetic peptide that preserves the receptor-binding region, to APP/PS1/ApoETR mice for 40 days resulted in a reduction of AD pathology and improved behavioral outcomes [169]. CN-105 was also tested in humans, and promising results were obtained. The intravenous infusion of CN-105 was well tolerated in healthy volunteers, with few and mild side effects [170-172]. Interestingly, CN-105 administration to patients with acute supratentorial intracerebral hemorrhage (1 mg/kg, every 6 h for 3 days after the acute event) resulted in an improved neurological outcome [172]. More recently, a randomized, blinded, placebo-controlled phase II sequential dose escalation trial was designed to evaluate perioperative administration of CN-105 in 201 adults (age ≥ 60 years) undergoing non-cardiac, non-neurological surgery (NCT03802396; Modulating ApoE Signalling to Reduce Brain Inflammation, Delirium, and Postoperative Cognitive Dysfunction - MARBLE) [173]. The study was completed in late 2024, and the results, although not yet available, are expected to be clinically relevant to AD. Indeed, the incidence of perioperative neurocognitive disorders, common complication in older adults, has shown to be associated with the ApoE4 genotype and possibly related to neuroinflammation, both common hallmarks of AD [174]. Have also investigated the possibility of preventing specific ApoE4-associated neuropathology by modifying its structure into an ApoE3-like structure, thus reducing self-aggregation and allowing proper lipidation. Moreover, considering that the ApoE4 genotype has been shown to affect the efficacy and safety of novel anti-Aβ immunotherapy approaches (*i.e*., amyloid-related imaging abnormalities (ARIA)) [175], the possibility of modifying ApoE4 structure into an ApoE3-like one might also impact the response to the currently available pharmacological approaches. In this regard, the phthalazinone derivative PH002, by interacting with the amino acidic residues arginine 61 and 199, crucial for ApoE4 specificity, was able to rescue the ApoE4-related impairment of neurite outgrowth in Neuro-2a cell line and dendritic spine development in primary murine neurons [176]. Importantly, PH002 treatment in human ApoE4 iPSC-derived neurons was able to dose-dependently ameliorate the detrimental effects of ApoE4 on Aβ secretion, GABAergic neuron degeneration, and tau phosphorylation [177]. Finally, two additional compounds, GIND-25 and GIND-105, were tested in cultured neurons to make ApoE4 an ApoE3-like protein [176, 178], providing a proof of concept that correcting the pathogenic conformation of ApoE4 to enhance its lipid-binding characteristics might represent a valid pharmacological approach in AD. Furthermore, the conversion of ApoE4 to ApoE3 expression using the CRISPR/Cas9 technique in human iPSC-derived astrocytes and neurons was sufficient to attenuate several AD-related phenotypes [179]. However, despite promising *in vitro* evidence, the actual clinical translatability of this approach appears challenging. Gene therapy approaches have also been investigated, aiming at overexpressing ApoE2 isoform in ApoE4 carriers using vector-based or liposomal delivery systems, following first experimental evidence that a single intracerebrovascular injection of adeno-associated viral (AAV) vectors delivering ApoE2 reduced Aβ deposition, prevented synapse loss, and attenuated neurite damage in ApoE4-expressing APP/PS1 mice [180, 181]. More recently, the administration of AAVrh.10hApoE2-HA (LX1001), an AAV vector containing the human ApoE2 cDNA sequence, in non-human primates successfully distributed ApoE2 in AD-relevant brain regions, without raising any specific safety concerns [182]. Consistently, a phase I-II open-label clinical trial is currently ongoing to evaluate the safety and efficacy of LX1001 in ApoE4 homozygous AD patients (NCT03634007). Similarly, brain-targeted liposomal delivery systems encoding ApoE2 plasmid DNA (pApoE2) have shown efficient brain transfection after intravenous injection in mice, without toxicity [183, 184]. Finally, in conjunction with pharmacological approaches aimed at correcting the pathological ApoE4-related structure, accumulating evidence suggests that anti-ApoE4 antibodies may represent an additional potential pharmacological approach for targeting non-lipidated ApoE4 in order to mitigate AD pathology. Among these, the antibody HAE-4, which selectively binds to non-lipidated ApoE4 (140-160 amino acid region) and the aggregated form of ApoE4 present in amyloid plaques, demonstrated significant reduction in Aβ deposition when administered *via* continuous intracerebral infusion (0.3 μg/h for 3 weeks) or through weekly intraperitoneal injections (50 mg/kg for 2 months) in APP/PS1-21/ApoE4 knock-in mice, also showing an efficient BBB crossing [185]. Notably, the intraperitoneal injection of HAE-4 (50 mg/Kg, 2 months) in a mouse model expressing human ApoE4 reduced Aβ plaque formation and cerebral amyloid angiopathy (CAA), together with the down-regulation of pro-inflammatory signalling in microglia and astrocytes [186]. Another ApoE4 targeting antibody, 7C11, was shown to reduce ApoE4-induced neurotoxicity *in vitro* and tau phosphorylation when administered to ApoE4 knock-in mice (50 mg/kg) [187]. Other similar approaches involving anti-ApoE4 monoclonal antibodies, such as 9D11, injected intracerebroventricularly in ApoE4 mice [188], and HJ6.3 injected intraperitoneally in APP/PS1 mice [189], led to a significant amelioration of cognitive dysfunction associated with reduced Aβ pathology and microglial activation. Although further research is needed to enhance antibody selectivity and optimize the safety and efficacy in humans, ApoE4 immunotherapy currently represents a valid potential therapeutic approach for AD. Together with ApoE4-targeting antibodies, the rationale for targeting ApoE4 through specific antisense oligonucleotides (ASO) has also been explored. As such, the intracerebral administration of ApoE4-ASO to APP/PS1 ApoE4 homozygous mice significantly decreased Aβ pathology when the treatment started prior to plaque deposition [190]. Similarly, the intracerebroventricular injection of ApoE ASO in the P301S/ ApoE4 mouse model of tauopathy significantly reduced ApoE4 levels, tau pathology, and neuroinflammation [191]. More recently, a CNS-targeted siRNA effectively silenced ApoE4 expression in adult 5XFAD mice, reducing Aβ burden without affecting circulating cholesterol levels [192].

##### 3.2.2.2 ApoA-I- and ApoJ-targeting Experimental Approaches

Beyond ApoE-targeting therapies, additional pharmacological targets and approaches have been proposed to counteract AD-related alterations in brain cholesterol transport. Among these, we have recently reported lower ApoA-I serum levels in AD patients compared to cognitively normal individuals [192]. Consistently, the overexpression of human ApoA-I has been associated with reduced neuroinflammation and improved cognitive performance in APP/PS1 mice [193], despite no evidence being available in humans yet. In addition, the chronic infusion of the natural occurring ApoA-I variant known as ApoA-I Milano, traditionally associated with lower cardiovascular disease risk as a consequence of its major efficiency in promoting cholesterol efflux [194], in APP23-transgenic aged and middle-aged AD mice led to a significant reduction in cerebral amyloid load and reduced neuroinflammatory markers [195, 196], thus paving the way for ApoA-I-targeting therapies in AD. However, as previously discussed, HDL-associated apolipoproteins have shown a limited BBB crossing ability, while small peptides, characterized by a higher BBB permeability, might be better suited for CNS-targeted therapy [2, 197]. In this regard, the systemic infusion of ApoA-I mimetic peptide 4F in AD mice was associated with increased Aβ clearance and improved cognitive functions [197, 198]. Unlike ApoE and ApoA-I-targeting therapies, ApoJ-related experimental approaches are currently less investigated, despite ApoJ's involvement in various physiological and pathological processes related to AD pathogenesis [199]. In this regard, the 2-week intraventricular infusion of the ApoJ mimetic peptide derived from residues (113-122), termed APOJ (113-122), in 5XFAD mice improved memory functions and lowered Aβ deposition and cerebral amyloid angiopathy [200].

##### 3.2.2.3 Reconstituted HDL Infusion

Several studies have also investigated whether the infusion of reconstituted HDL (rHDL) might represent a possible pharmacological strategy to counteract neurodegeneration. In this regard, the 4-week infusion of rHDL containing lipidated ApoE3 was associated with boosted cognitive performance and Aβ clearance in AD mice [201]. Similarly, a single acute intravenous injection of reconstituted HDL containing phosphatidylcholine and ApoA-I significantly reduced cerebral amyloidosis in APP/PS1 transgenic mice; this effect, however, was no longer apparent after 4 consecutive once-weekly injections, suggesting that the beneficial effects diminish as rHDL is cleared from the body [202]. Finally, the intravenous administration of rHDL-ApoJ nanoparticles in old APP23 AD mice, known to mediate higher cholesterol efflux in cultured macrophages, was shown to reach the CNS effectively [203] and reduce insoluble Aβ fibrils and cerebral amyloidosis, possibly due to the upregulation of phagocytic pathways in myeloid cells [204].

##### 3.2.2.4 HDL-like Accessory Proteins

Despite the increasing interest and extensive research on the topic, there are still no validated dietary or pharmacological approaches that can effectively modulate PON1, the only HDL-accessory protein likely associated with HDL-like particles.

### 3.3 Neuronal Cholesterol Internalization and Cholesterol Processing

#### 3.3.1 Modulation of Cholesterol Internalization into Neurons

As already mentioned, neuronal cholesterol supply is ensured through the delivery of cholesterol to neurons *via* specific ApoE-interacting receptors, such as LDLR, ApoER2, and LRP1. An impairment of this last step of brain cholesterol transport is indeed associated with neurodegenerative disorders, particularly AD [83], and its mediators are thus considered potential pharmacological targets that could contribute to halting the disease.

##### 3.3.1.1 LDLR Targeting Approaches

An *in vivo* study using a mouse model overexpressing LDLR showed an increase in Aβ clearance and reduced cerebral deposition. Based on this observation, the researchers performed a phenotypic screening of small molecules targeting regulators of LDLR expression in glial cells that could represent new therapeutics against neurodegeneration, despite no evidence is available yet [205].

##### 3.3.1.2 ApoER2-targeting Approaches

Both natural and newly synthesized compounds targeting ApoER2 are under investigation for the treatment of neurodegeneration. Indeed, in the study carried out by Feng *et al*., carnosic acid, already known for its neuroprotective and neurotrophic functions, was demonstrated to reverse the negative effect of ApoE4 on ApoER2 recycling by increasing the activation of the reelin signalling pathway. Moreover, treatment with carnosic acid was also able to restore neurite growth, which had been altered by the presence of ApoE4, suggesting its potential use in attenuating ApoE4-associated AD [206].

##### 3.3.1.3 LRP1-targeting Approaches

A large body of evidence points to LRP1 as a possible pharmacological target for the treatment of AD, as it is strongly implicated in the regulation of Aβ peptide production and clearance from the CNS to the periphery [98, 207]. A plant secondary metabolite compound, ginsenoside compound K, has been demonstrated to enhance LRP1 expression in a model of mouse microglia, thus contributing to the amelioration of inflammation by reducing cytokine production and NF-kB activation induced by Aβ oligomers [208]. The positive effects of enhanced LRP1 expression were also demonstrated in another *in vitro* study, where endothelial cells treated with pioglitazone significantly attenuated Aβ deposition through increased LRP1 expression. These results were corroborated by *in vivo* experiments, which demonstrated an improvement in the cognitive performance of a mouse model of senescence after treatment with pioglitazone at a low dose (2 mg/Kg/day), accompanied by an increase in LRP1 expression and a reduction in Aβ deposition [209].

##### 3.3.1.4 PCSK9-targeting Approaches

Based on the growing evidence suggesting a clear involvement of PCSK9 in brain cholesterol transport and AD pathophysiology, its pharmacological inhibition has been considered as a potential therapeutic strategy against the disease [210]. Several approaches have been tested in preclinical models, including monoclonal antibodies targeting PCSK9, as well as small molecules, both synthetic and natural, that either inhibit PCSK9 expression or interfere with its interaction with target receptors [211, 214]. However, most of the studies have been conducted on experimental models involving high-fat-induced cognitive dysfunction, showing overall ameliorating effects [212-215]. More interesting has been the study by Mazura *et al*., where the treatment of a specific mouse model of AD with FDA-approved antibodies against PCSK9 led to an improvement in cognitive performance, along with a reduction in Aβ pathology [216]. This effect disappeared in mice lacking the receptor LRP1 selectively at the BBB, confirming a peripheral effect of these PCSK9 inhibitors. Preserving the expression of LRP1 on endothelial cells of the BBB could favour LRP1-mediated Aβ clearance from the brain. This explanation is consistent with the incapability of monoclonal antibodies to directly access the brain, at least through an intact BBB. In this regard, we have previously identified some small and lipophilic molecules with potent inhibitory activity on PCSK9 expression and secretion, which are well-tolerated *in vivo* [217], and are being further tested *in vitro* and in mouse models as a potential therapeutic strategy for AD, with high clinical relevance. In this regard, it is worth noting that in humans, the monoclonal antibodies evolocumab and alirocumab were initially suspected of inducing adverse cognitive effects instead of beneficial ones [101]. However, the subsequent results of several trials, including the EBBINGHAUS study [218], as well as a parent study with a longer follow-up time (median of 5.1 years) [219], did not reveal any cognitive impairment associated with PCSK9 inhibitors. Interestingly, in the Real World-MEMOGAL Study, the use of such antibodies was instead associated with an improvement in a specific memory test, namely the delayed recall memory [220].

##### 3.3.1.5 IDOL-targeting Approaches

The E3 ubiquitin ligase IDOL is a known LDLR- and ApoER2-targeting E3 ubiquitin ligase that acts in combination with PCSK9 by post-transcriptionally modifying its expression, thereby affecting cerebral lipid metabolism [109]. Consequently, recent experimental evidence has explored the potential inhibition of cerebral IDOL as a therapeutic strategy for AD. Choi and colleagues reported IDOL genetic ablation in APP/PSEN1 mice to be associated with increased brain LDLR expression, reduced Aβ plaque burden, and decreased neuroinflammation [221], thus reinforcing IDOL-mediated activity as a potential therapeutic intervention in AD. Consistently, the administration of an ASO to reduce cerebral IDOL expression in APP/PS1 mice was associated with decreased Aβ pathology as well as improved spatial learning and memory in the presence of an upregulation of lysosomal and phagocytic pathways in hippocampal microglia [222]. Altogether, this evidence suggests that cerebral IDOL reduction might be further investigated as a potential therapeutic strategy for AD treatment.

#### 3.3.2 Modulation of Neuronal Cholesterol Processing

Recent insights into the modulation of CYP46A1 and hydroxysterols generation have shed light on potential pharmacological strategies for AD. Supporting this hypothesis, the injection of an adeno-associated vector encoding for CYP46A1 in the cortico-hippocampal areas of APP/PS1 mice was associated with a remarkable reduction of amyloidosis, along with an improvement in spatial memory [223]. Consistently, hippocampal CYP46A1 mRNA inhibition in APP23 Tg mice led to cognitive deficits, likely related to increased hippocampal apoptosis, tau hyperphosphorylation, APP recruitment within lipid rafts, and Aβ peptide generation [224]. As such, the administration of a small dose (0.1 mg/kg body weight/day) of the anti-HIV drug efavirenz [225, 226], able to activate CYP46A1, in transgenic 5XFAD mice was shown to enhance retinal cholesterol turnover, associated with a reduction in vascular lesions and Aβ plaques accumulation [227]. Consistently, other pre-clinical studies have demonstrated that enhancing CYP46A1 activity through efavirenz led to reduced Aβ accumulation and amelioration of cognitive deficits in mouse models of AD [228-231], possibly due to the modulation of several processes secondary to changes in sterol fluxes [232]. This aligns with findings that increasing 24HC levels through CYP46A1 activation can positively influence synaptic activity and mitigate neuroinflammatory responses associated with AD [233]. Accordingly, a randomized controlled clinical trial (NCT03706885) has been conducted as a proof-of-concept to support rationale of efavirenz treatment (50 mg and 200 mg, for 20 weeks) in patients with early AD, observing, despite the small number of patients recruited, a significant increase in plasma 24HC levels from baseline, that, however, did not correlate with the most relevant AD CSF biomarkers. Interestingly, the Montreal Cognitive Assessment (MoCA) score was increased by 3 points in patients receiving the 50 mg dose [234]. This evidence, however, requires a more comprehensive and in-depth evaluation to further confirm the possibility of repurposing efavirenz as a candidate drug for targeting AD biomarkers and clinical symptomatology [226].

## CONCLUSION

In the brain, cholesterol levels are tightly regulated. One peculiar aspect of cerebral cholesterol homeostasis maintenance is the transport of cholesterol from astrocytes to neurons, which is essential to guarantee neuronal functions, such as synaptogenesis, neurite outgrowth, and the repair of damaged membranes. This process occurs through the complex interplay among membrane transporters, lipoprotein particles, and relative associated proteins, as well as receptors, whose dysregulation has been associated with neurodegeneration and AD. Based on this evidence, this review has examined the individual factors involved in brain cholesterol transport, their alterations in AD, and their role as potential targets for novel pharmacological approaches. Although the majority of the proposed strategies are still at the pre-clinical stage, the collected clinical data suggest that targeting the efflux of cholesterol from astrocytes or glial cells may represent one of the most promising approaches to counteract AD. In this regard, the ApoE mimetic peptide CN-105 has reached phase II for the treatment of perioperative neurocognitive disorders, raising the possibility of being tested as a novel therapeutic agent for AD. In addition, enhancing the last step of brain cholesterol transport, the delivery of cholesterol to neurons, may also be considered a valid strategy to counteract AD. This may be achieved by targeting PCSK9, by means of small molecules accessing the CNS that are now under investigation in pre-clinical stage, with potential outstanding clinical relevance. Confirmation of these preliminary observations in further studies may pave the way for the development of novel therapeutic opportunities based on the lipid hypothesis for AD pathogenesis, as an alternative to current treatments that still lack convincing efficacy.

## Figures and Tables

**Fig. (1):**
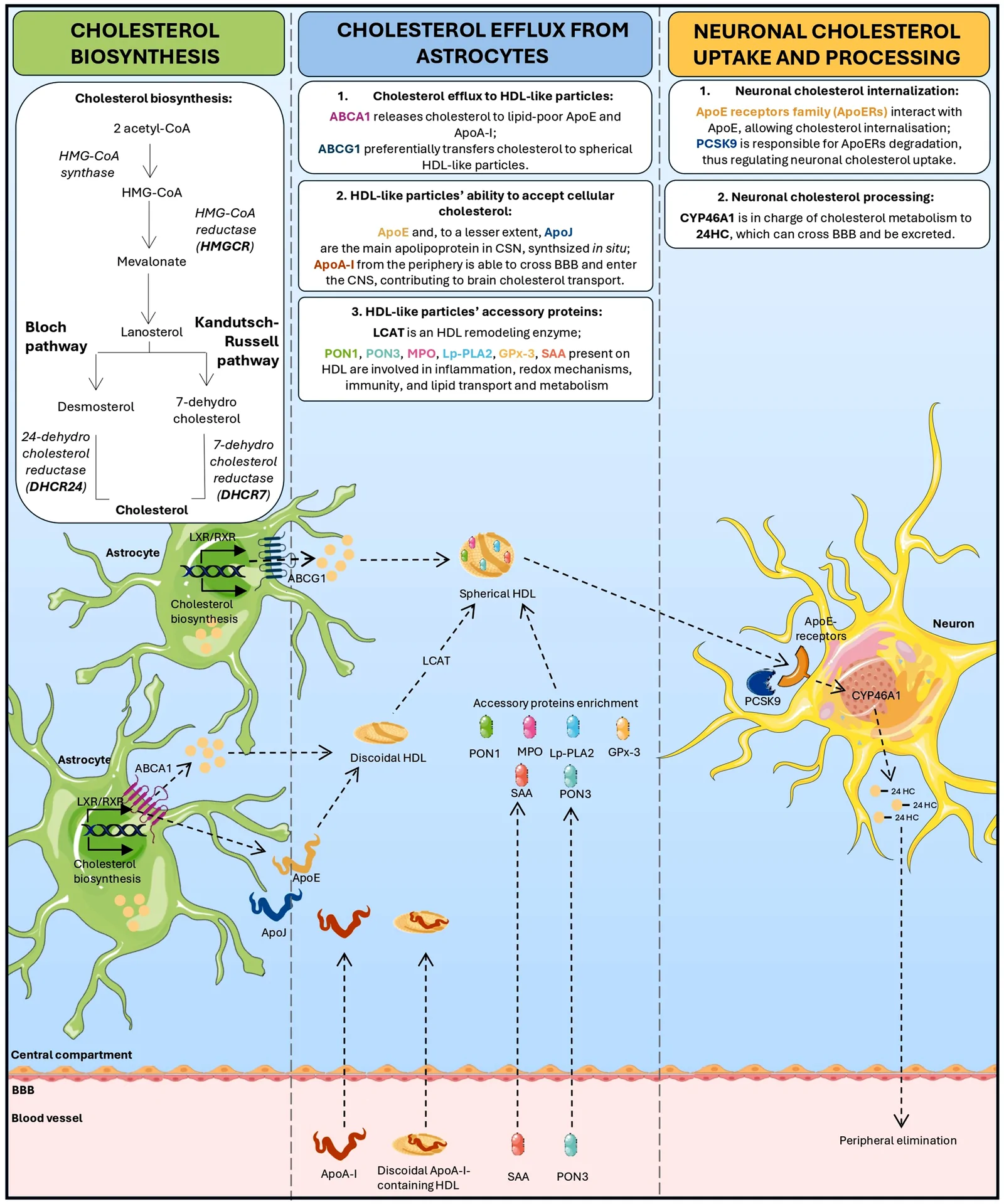
Representation of brain cholesterol transport. Cerebral cholesterol homeostasis in the CNS is maintained by the interplay of several mediators, which act in three different steps of cholesterol transport: cholesterol biosynthesis, cholesterol efflux from astrocytes, and neuronal cholesterol uptake and processing. The picture has been created by combining images from Smart Sevier Medical Art (https://smart.servier.com) accessed on 09.06.25. Sevier Medical Art by Sevier is licensed under a Creative Commons Attribution 3.0 Unported License (https://creativecommons.org/licenses/by/3.0/). **Abbreviations**: ABCA1, ATP-binding cassette transporter A1; ABCG1, ATP-binding cassette transporter G1; Acetyl-CoA, Acetyl-coenzyme A; ApoA-I, Apolipoprotein A-I; ApoE, Apolipoprotein E; ApoJ, Apolipoprotein J; ApoER2, Apolipoprotein E receptor 2; BBB, Blood-brain barrier; CNS, Central nervous system; CYP46A1, Cytochrome P450 46A1; DHCR24, 24-dehydrocholesterol reductase; GPx-3, Glutathione peroxidase 3; HDL, High-density lipoprotein; 24HC, 24-hydroxycholesterol; HMG-CoA, 3-hydroxy-3-methylglutaryl-coenzyme A; HMGCR, 3-hydroxy-3-methylglutaryl-coenzyme A reductase; LCAT, Lecithin:cholesterol acyltransferase; Lp-PLA2, Lipoprotein-associated phospholipase A2; LXR, Liver X receptor; MPO, Myeloperoxidase; PCSK9, Proprotein convertase subtilisin/kexin type 9; PON1, Paraoxonase 1; PON3, Paraoxonase 3; RXR, Retinoid X receptor; SAA, Serum amyloid A*.*

**Table 1:** Main alterations in brain cholesterol transport associated with Alzheimer’s disease.

**Target**	**Alterations Observed in AD**	**References**
**1.1. Alterations in Endogenous Cholesterol Biosynthesis**		
**HMGCR**	HMGCR expression was reduced in the human brain tissue of AD patients	[17]
	HMGCR gene polymorphism (rs3846662) was associated with lower AD risk	[18, 19]
**DHCR24**	DHCR24 inhibition was associated with cholesterol depletion, reduced neuronal plasticity and functionality, increased Aβ accumulation, and reduced cognitive performance in a rat model	[21]
	Cholesterol overload led to neuronal apoptosis and DHCR24 downregulation	[22]
	Downregulation of DHCR24 in the hippocampus of HFD mice caused brain injury	[22]
**1.2. Alterations in Cholesterol Efflux from Astrocytes**		
**1.2.1. Alteration in Cellular Ability to Release Cholesterol**		
**ABCA1**	ABCA1 trapping in the lysosome caused cellular senescence and cholesterol accumulation in the ApoE4-TR mice model	[28]
	Poor ABCA1 recycling in AD conditions caused reduced ApoE levels and poor ApoE lipidation in primary mouse astrocytes and APP23 mice	[29, 33]
	ABCA1 suppressed APP proteolysis, reducing Aβ deposition in a mouse model of AD	[33]
	ABCA1 function was affected by the presence of Aβ deposition, reducing cholesterol efflux in primary mouse brain cells and human astrocytes	[36, 130]
**ABCG1**	ABCG1 reduced Aβ production in HEK293 cells, stably expressing human APP695 containing the Swedish mutation	[31]
**ABCA7**	ABCA7 was found to be involved in Aβ aggregation and deposition, and ABCA7 SNPs were associated with an increased risk of AD	[38]
**ABCG4**	ABCG4 inhibited γ-secretase activity and Aβ production in HEK293 cells, stably expressing human APP695 containing the Swedish mutation	[39]
**1.2.2. Alteration in HDL-like Particles' Ability to Accept Cellular Cholesterol**		
**HDL CEC**	CSF ABCA1- and ABCG1-mediated CEC was reduced in MCI and AD patients compared to control subjects	[15, 43-45]
**ApoE**	ApoE4 lipidation was reduced compared to ApoE3 and ApoE2, leading to a lower cholesterol efflux promotion in mice and human brain cells and in CSF samples of AD individuals	[47-50]
**ApoA-I**	Deficiency of ApoA-I led to increased levels of Aβ deposition and inflammation in APP/PS1 mice	[51]
**ApoJ**	Lower levels of ApoJ were associated with reduced cholesterol efflux capacity in MCI patients	[52]
**1.2.3. HDL-like Particles’ Accessory Proteins**		
**PON1**	PON1 prevented oxidative stress and lipid peroxidation in Tg2576 and 3xTg-AD mice	[78, 79]
	Lower PON1 levels and activity were found in 3xTg-AD mice and AD patients compared to cognitively healthy subjects	[79, 81]
**1.3. Alteration in Neuronal Cholesterol Internalization and Processing**		
**1.3.1. Alteration in Cholesterol Internalization into Neurons**		
**LDLR**	The lack of LDLR was associated with memory impairment in murine models of mild and severe AD	[84-88]
	Genetic mutation of LDLR (variant rs140241383) was associated with the risk of AD	[89, 90]
	LDLR recycling was affected by ApoE4 presence in AD in *in silico* studies	[91]
**ApoER2**	Disruption of the ApoER2 pathway increased p-tau formation in post-mortem brain tissues	[92]
	Alternative splicing forms of *ApoER2* were associated with different AD risks	[93]
	ApoER2 levels were lower in AD patients compared to control subjects	[94]
**Reelin**	Alteration in reelin fragments was observed in the CSF of AD patients compared to controls	[94]
	Gain-of-function mutation of *RELN* was associated with resilience against AD	[96]
**LRP1**	LRP1 contributed to Aβ clearance across BBB, as demonstrated in *APP^Sw/0^* mice and tissue sections derived from AD patients	[97]
	LRP1 silencing led to Aβ deposition, neuroinflammation, synaptic loss, and reduced cognitive performance in AD mouse models	[98]
**PCSK9**	Higher CSF PCSK9 levels were found in AD compared to non-AD subjects, particularly in ApoE4-carriers	[102, 103]
	Elevated PCSK9 mRNA and protein levels were observed in the frontal cortex of AD patients	[104]
	PCSK9 reduced ApoE-mediated neuronal cholesterol uptake and intracellular cholesterol in SH-SY5Y neuroblastoma cells	[105]
	PCSK9 exacerbated both Aβ-induced cytotoxicity (SH-SY5Y neuroblastoma cells) and inflammation (U373 astrocytoma cells)	[105, 106]
	Genetic ablation of PCSK9 led to the amelioration of cognitive performance, Aβ deposition, and neuroinflammation in 5XFAD mice	[106]
	Neuron-specific PCSK9 deletion led to an impairment in cognitive function and alterations in hippocampal synapse morphology in primary mouse hippocampal cells	[107]
**IDOL**	IDOL controlled brain LDL receptor expression, ApoE clearance, and Aβ amyloidosis in APP/PS1 mice	[110, 111]
**1.3.2. Alteration in Neuronal Cholesterol Processing**		
**CYP46A1**	CYP46A1 expression levels were lower in AD patients compared to controls	[116, 117, 119]
**24HC**	Increased levels of 24HC correlated with neuronal damage and AD, when AD patients and control subjects were compared	[103, 117, 118, 120-124]
	24HC reduced intraneuronal accumulation of hyperphosphorylated tau protein in SK-N-BE neuroblastoma cells and in hTau mice	[126, 127]
	24HC reduced Aβ production, inhibiting APP in human neuroblastoma SH-SY5Y cells, CHO cells stably expressing human APP, and primary mouse astrocytes	[128, 129]

**Abbreviations**: ABCA1, ATP-binding cassette transporter A1; ABCA7, ATP-binding cassette transporter A7; ABCG1, ATP-binding cassette transporter G1; ABCG4, ATP-binding cassette transporter G4; Aβ, Amyloid beta; AD, Alzheimer’s disease; APP, Amyloid precursor protein; ApoA-I, Apolipoprotein A-I; ApoE, Apolipoprotein E; ApoJ, Apolipoprotein J; ApoER2, Apolipoprotein E receptor 2; CEC, Cholesterol efflux capacity; BBB, Blood-brain barrier; CSF, Cerebrospinal fluid; DHCR24, 24-dehydrocholesterol reductase; CYP46A1, Cytochrome P450 46A1; HDF, High-fat diet; HDL, High-density lipoprotein; 24HC, 24-hydroxycholesterol; HMGCR, 3-hydroxy-3-methylglutaryl-CoA reductase; IDOL, Inducible degrader of LDL receptor; LDL, Low-density lipoprotein; LDLR, Low-density lipoprotein receptor; LRP1, Low-density lipoprotein receptor-related protein 1; MCI, Mild cognitive impairment; PCSK9, Proprotein convertase subtilisin/kexin type 9; PON1, Paraoxonase 1; SNPs, Single nucleotide polymorphisms.

## LIST OF ABBREVIATIONS

AD: Alzheimer’s Disease
ApoE: Apolipoprotein E
APP: Aβ Precursor Protein
Aβ: Amyloid β
BBB: Blood-brain Barrier
BDNF: Brain-derived Neurotrophic Factor
CETP: Cholesteryl Ester Transfer Protein
CNS: Central Nervous System
CSF: Cerebrospinal Fluid
LCAT: Lecithin Cholesterol Acyltransferase
MCI: Mild Cognitive Impairment
MPO: Myeloperoxidase
PCSK9: Proprotein Convertase Subtilisin/Kexin Type 9
PLTP: Phospholipid Transfer Protein
SNPs: Single-nucleotide Polymorphisms
